# The effect of patient death on medical students in the emergency department

**DOI:** 10.1186/s12909-017-0945-9

**Published:** 2017-07-10

**Authors:** Nicholas J. Batley, Rinad Bakhti, Ali Chami, Elsy Jabbour, Rana Bachir, Christopher El Khuri, Afif J. Mufarrij

**Affiliations:** Department of Emergency Medicine, American University of Beirut Medical Center, P.O.Box - 11-0236 Riad El Solh, Beirut, 1107 2020 Lebanon

**Keywords:** Medical students, Patient death, Emotional reaction, Emergency department, Inpatient setting, Patient interaction, Coping mechanisms

## Abstract

**Background:**

The emotional consequences of patient deaths on physicians have been studied in a variety of medical settings. Reactions to patient death include distress, guilt, and grief. Comparatively, there are few studies on the effects of patient death on physicians and residents in the Emergency Department (ED). The ED setting is considered unique for having more sudden deaths that likely include the young and previously healthy and expectations for the clinician to return to a dynamic work environment. To date, no studies have looked at the effects of patient deaths on the more vulnerable population of medical students in the ED. This study examined aspects of patient deaths in the ED that most strongly influence students’ reactions while comparing it to those of an inpatient setting.

**Methods:**

Semi-structured qualitative interviews were carried out with a total of 16 medical students from the American University of Beirut, Medical Center in Lebanon who had recently encountered a patient death in the ED. Questions included their reaction to the death, interaction with patients and their family members, the response of the medical team, and coping mechanisms adopted.

**Results:**

The analysis revealed the following as determinant factors of student reaction to patient death: context of death; including age of patient, expectation of death, first death experience, relating patient death to personal deaths, and extent of interaction with patient and family members. Importantly, deaths in an inpatient setting were judged as more impactful than ED deaths. ED deaths, however, were especially powerful when a trauma case was deemed physically disturbing and cases in which family reactions were emotionally moving.

**Conclusion:**

The study demonstrates that students’ emotional reactions differ as a function of the setting (surprise and shock in the ED versus sadness and grief in an inpatient setting). Debriefing and counseling sessions on ED deaths may benefit from this distinction.

**Electronic supplementary material:**

The online version of this article (doi:10.1186/s12909-017-0945-9) contains supplementary material, which is available to authorized users.

## Background

Medical professionals dedicate their lives to the treatment of patients in an attempt to cure, all the while knowing that any cure is temporary. Despite being a prevalent experience, a relatively small number of studies have shed light on death’s emotional effects on physicians [1]. Perhaps unsurprisingly, greater exposure to death was found to be strongly associated with greater work-related stress [2]. In one study, 61% of physicians interviewed reported that their most memorable patient death remains emotionally distressing [3]. Cases that elicit strong emotional reactions include: patients with whom the physician feels an interpersonal connection, those who are not cured by standard treatment, younger patients, and deaths that lack dignity (e.g., futile resuscitation) [4]. After a recent patient death, physicians report feelings of numbness, feeling upset when thinking about the patient [4], guilt, and stress [5].

### Physicians in the emergency department

Studies addressing the effects of death on physicians in the emergency department (ED) are similarly minimal. It is recognized, however, that the ED is a unique setting: there is generally no previously established relationship between the patient and the physician, deaths are likely to be sudden, and could include the young and previously healthy. Moreover, after a death, ED physicians are often expected to function in a dynamic work environment almost immediately, care for other patients, and handle a grieving family [6]. Deaths that are characterized as shocking and unexpected are known to lead to more distress and psychological trauma than natural and expected deaths [7].

One study found that 28% reported they sometimes considered quitting their jobs, 32% changing professions, and 14% seeking counseling [8]. ED physicians also experienced insomnia and fatigue as well as emotional responses of sadness and disappointment. The age of the patient, cause of death, and presence of family played a role in the intensity of emotion [8].

### Residents in the ED

The effect of patient death may vary depending on one’s level of training. For example, residents perceive patient deaths as more disturbing compared to attending physicians and are more likely to rate a death as shocking or unexpected [7]. The effect of patient death on ED residents specifically is understudied [6]. One study reported that 49% expressed distress or fear in reaction to patient death [9]. Nearly 12% met full criteria for Post-Traumatic Stress Disorder, while 30% met at least one symptom in each of the three symptom categories: re-experiencing, avoidance, and arousal [9].

### Medical students in an inpatient setting

A qualitative study investigating the emotional reactions of 32 students to patient deaths during their internal medicine rotation found that 57% considered the death emotionally powerful [10]. In response to patient death, 73.1% reported crying during clinical rotation [11]. Nearly half of medical students stated feeling unprepared to handle emotional reactions to patient death or help grieving families [12] and found dealing with the family of the dying as difficult as dealing with the dying [13]. Another study demonstrated that students’ most common emotional reactions included sadness, whereas the most common coping mechanism was transforming death into a learning experience for those experiencing patient death the first time [14]. Importantly, reaction to patient death varied depending on the age of the patient (young vs. old), whether it was expected or unexpected, and difficult or peaceful.

### Aims of study

To our knowledge, no studies to date have examined the effect of patient death on medical students in the unique setting of the ED. This study aimed to assess students’ reactions to patient deaths in the ED compared to an inpatient setting, including aspects of death that generated the most powerful emotional responses, coping mechanisms, and whether or not counseling before the ED rotation or debriefing after a death would be deemed useful. We hypothesize that deaths in the ED may produce different reactions in students than deaths in the inpatient setting based on several factors that distinguish the ED: more cases of trauma, patients could include the young and previously healthy, generally shorter interactions with patients and their families, as well as the ED’s quick and fast-paced environment which may provide less opportunity to reflect on a patient death. The unique effects of patient death in the ED are potentially better understood when contrasted with the inpatient setting. Ultimately, understanding medical students’ experiences in the ED may allow for better interventions to help students cope with potentially traumatic experiences.

## Method

### Procedures

Qualitative data collection was carried out via semi-structured, in-depth interviews that allowed students to tell their individual stories about the deceased patients they witnessed (Additional file 1). This methodology is suitable to understand the beliefs, knowledge and experience of the participants [15, 16].

### Study setting and participants

- We surveyed 16 four-year medical students studying at the American University of Beirut (AUB) between 2014 and 2015. All students were contacted by phone by the research assistant and asked whether they had experienced a patient death. Those who had been exposed to a patient death were asked whether they would agree to participate in the study. None of the students who were approached refused to participate. A research assistant contacted them during or within three months after their rotation in the ED. They were asked to participate in 30 min interviews of open-ended and semi-structured questions about their experience with patient death in the ED and the inpatient setting. We stopped recruiting students when we reached data saturation. That is, when participant responses became redundant and no new codes or patterns were detected. The study received ethical approval from the institutional review board at AUB with the requirement that the students consent prior to participation. At the American University of Beirut Medical Center, fourth year medical students are senior students, in their fourth of four years and second year of clinical clerkships. Although all the students are bilingual the Arabic language was not used and all interviews were conducted in English.

### Interviews with students

Four researchers conducted 30 min interviews with the students. One has a master’s degree in public health and is a trained qualitative interviewer. The other three have medical degrees and underwent rigorous training: doing mock interviews and receiving feedback regarding their probing methods.

The semi-structured questionnaire consisted of four parts:

1. Demographic characteristics (age, gender)
2. Death in an inpatient setting
3. Death in the ED

Students were asked to describe the course of the patient’s illness and death, their reaction to the death, their involvement in the process of giving care, their interaction with the family members of the patient, the reaction of the medical team and how their behavior affected the student, and how the relationship to other patients changed since they experienced the death.

4- Pre-rotation counseling versus debriefing: the students were asked about potentially helpful ways to cope with the emotions generated after a patient’s death. They were asked if a counseling session before the rotation aimed at preparing them for what they might experience would be helpful, versus a brief debriefing session by the ED attending immediately after a death.

Semi-structured probes were used to acquire more details about the student’s experience. Particular issues included the cause of death, whether the student did CPR or administered any medication to the patient, the closeness of the student-patient relationship, how the student was affected emotionally by the death, and with whom the medical student shared their experience to seek support.

### Analysis

Informed consent was obtained from all participants. Interviews were audio taped and subsequently transcribed verbatim.

Analysts included one medical doctor, a public health practitioner and a psychologist. The psychologist was involved in the qualitative data analysis but did not conduct any interview. This minimized the influence of biases on the data. The medical doctor and the psychologist coded the data and identified themes while the public health practitioner served as an independent verifier.

Thematic content analysis, a method of identifying, analyzing, and reporting patterns or themes within the data, was used to examine the transcripts [17].

Themes or patterns within the data were identified in an inductive or bottom up way. The qualitative data analysis was then based on grounded theory to identify recurrent themes and emergent patterns. Grounded theory is a method of identifying analytical themes as they emerge from the data [16] and then constructing theories that are grounded in the data itself. Unlike the traditional model of research in which the researcher determines whether or not the theory applies to the phenomenon under study after collecting the data, grounded theory is a systematic methodology of theory built upon the categories generated by reviewing and coding the collected data. The grounded theory method is implemented in 4 steps: 1) verbatim read-through of every transcript 2) pinpointing potential themes 3) comparing themes, and lastly 4) constructing a theoretical framework.

Grounded theory was helpful in allowing for an exploration of emerging themes in the participants’ responses that may be specific and unique to an ED setting in a Lebanese and Middle Eastern context. A major contributing factor to emotional reactions to death, having an ongoing relationship with patients, is a distinctive feature that is notably lacking in the ED. Since ED encounters are often brief, with differing patient and family dynamics exploring the factors contributing to student reactions was the aim of this study. It is unclear whether these distinctive features of the ED serve as protective or harmful factors when confronted with patient death [6].

Qualitative data analysis consisted mainly of open coding (the process of sweeping through the data), axial coding (the process of sorting the codes into groups) and selective coding (the process of developing minor and major categories) [16].

The analysis proceeded through six stages:

1. In order to get a good sense of the data and to be familiar with it, the research team read through all the interviews.
2. Brief notes were included in the margin about the relevant information noticed.
3. Investigators developed a coding scheme after several meetings during which they discussed the appropriateness of each code and resolved disagreements through consensus.
4. A list of codes was created from the different types of information found; all codes were brought together in a spreadsheet to better manage the data.
5. The codes were linked into themes and subthemes.
6. The research team revisited the data multiple times, reviewing its categorization until the team felt confident that the themes and subthemes used to summarize the findings are a truthful and accurate reflection of the data.

## Results

The sample consisted of 16 students, 10 females (62.5%) and 6 males (37.5%), with a mean age of 23.7. The following themes emerged: context of death, interaction with patient and family, assessment of medical team, psychological consequences and coping mechanisms. Themes are discussed in relation to both the ED and the inpatient setting. Additionally, participant quotes are displayed [18]. The patient illnesses encountered in the ED and inpatient setting are presented in a table (Table 1).

**Table 1:** List of illnesses in the ED and inpatient setting

Type of illness in the ED	Type of illness in the inpatient setting
Cardiac arrest	Respiratory failure
Cardiac arrest	Cancer
Cardiac arrest	Cancer
Trauma	Diabetes
Sepsis	Renal failure
Cardiac arrest	Cancer
Burn	Cancer
Cardiac arrest	Palliative patient
Cardiac arrest	Pneumonia
Cardiac arrest	Cancer
Cardiac arrest	Renal failure
Trauma	Cancer
Trauma	Cancer
Suicide	Bradycardia
Cardiac arrest	Cystic Fibrosis
Trauma	Cystic Fibrosis

### Context of death

Students were asked about the contextual details surrounding their experienced deaths. These included the patient’s age, expectation of death, if it was the student’s first death, and whether or not the student related the patient death to personal experiences with death. In general, students reported a ‘sudden’ and ‘unexpected’ death when talking about deaths in the ED. By contrast, deaths in the inpatient setting were more likely to be described as ‘expected.’ Death of the young was viewed as unexpected whereas death of the old was expected. As a result, in a few cases, deaths in the ED were labeled as ‘expected’ when older patients were involved. Generally, deaths that are unexpected and involve the young were described as more emotionally moving. In some cases, experiences of first deaths appeared to be more impactful:

*“Yeah I witnessed a couple more but I wasn’t as emotional [as the first time]. I cried a couple more times. Towards the end of the year, I stopped crying.”*

*“It was the first time I saw death … I thought about the family because the death was unexpected, so I started to relate it to my family, I thought about death, the idea of death.”*

Relating death to personal experiences, including thinking about death of relatives as well as thinking about death generally, increased students’ emotional reaction, as illustrated in the second quote and the following:

*“I noticed mainly that it's a matter of age, 40s, my parent’s age, I’d be devastated.”*

*“She was already terminal… but you relate more to this. You start thinking of your grandma and your aunt.”*

### Interaction with patient and family

In relation to student interaction with patients, subthemes were characterized as active involvement and passive or minimal interaction. Greater interaction generally led to greater emotional investment. Regarding active involvement with patients in the inpatient setting, students’ behavior included: being caring, fighting for the patient, emotional attachment, closeness, and being there for the patient and family. One student says of a patient on the floor:

*“…Once I enter the room I just change everything in my mind because I know I care about them”.*

In the inpatient setting, students’ interaction with the patients was noticeably more extensive. Interaction with patients in the ED, on the other hand, was described in many cases as minimal, restricted to performing CPR, and observing. This, in turn, led to a less pronounced emotional reaction:

*“Well it’s because first of all, you know the patient. You’ve talked to them more. I had zero interactions with the ones in the ER or with their families. On the floor you’re more involved. You’ve talked to them; you’ve talked to their families you’ve followed them for weeks.”*

In general, greater interaction with the family of the deceased was associated with a stronger emotional reaction. A similar trend was detected whereby family interactions are more extensive and heavier in the inpatient setting when compared to the ED. Specifically, students in the inpatient setting described their interaction with the family as supportive, empathetic, and many ‘got to know the family very well.’

*“I got to know the family. I got to know his son, his wife, they spent all day there, every day and I spent all day there, every day.”*

By contrast, in many cases, family interactions within the ED were minimal. Nevertheless, cases in the ED where the family reaction to the death was witnessed also triggered emotional reactions:

*“They were shocked, they were crying. It was overwhelming. I had tears in my eyes again.”*

*“Seeing her [the wife] cry was more disturbing than the patient’s death.”*

The experience of accompanying attending physicians as they inform the family of death was also particularly stressful and confusing:

*“I did not know how to phrase it, how my expressions should be…”.*

### Assessment of medical team

When commenting on the behavior of the emergency department team, students rarely mentioned witnessing emotional reactions to patient death. In cases where they were raised, students reported no emotional reaction from the team and even a sense of detachment:

*“There was really no emotion, I felt.”*

*“I didn’t have any reaction from the rest of the team concerning the emotion.”*

In one case, a student assumes the attending was sad, but was unsure since no direct discussion took place:

*“I did not discuss that with them directly, I did not ask them how would they feel … they seemed sad but I can’t tell for sure.”*

The team’s behavior was instead most commonly described as professional, organized, systematic, calm, and efficient in both the ED and inpatient setting. This was regarded as a positive influence:

*“It affected me positively in the way they completely forgot about everything going on and were focused on the patient.”*

*“I realized that medical-wise, it was business as usual…”.*

### Psychological consequences and coping mechanisms

The psychological consequences of patient death are divided into three broad categories: emotional, physical and cognitive reactions. A wide range of emotional reactions was expressed in the inpatient setting including: sadness, helplessness, grief, disappointment, sympathy, crying, feeling disturbed, emotional, heartbroken, irritated, overwhelmed, traumatized, upset and anxious:

*“It was stressful. We did cry...The guys were bummed. 3 girls started crying for like 20 minutes.”*

In the ED, emotional reactions were of a different nature including: frustration, shock, powerlessness, confusion, surprise, and being affected by the imagery:

*“Till now I still don’t understand what happened. It’s more shocking, because nothing happened, and he had nothing… I just felt a bit more powerless.”*

*“That impacted me a lot. Just seeing the expression or lack of expression on her face… She had a blank look on her face. Her hands and arms were hyperextended at the elbow. The thighs were twisted... it was a very graphic scene… I still have a very vivid image of her in my head.”*

Notably however, four students reported being unaffected and regarded the death as part of the job. This occurred more so in the ED than in the inpatient setting. In the ED setting, not being involved in the care of the patient for a lengthy period of time helped students view the incident as an ‘experience on the job’, improved their ability to detach and lessened the overall impact.

*“So I was just, I don’t know, practicing CPR. That’s it. I wasn’t feeling anything. I wasn’t affected by the death… I was just learning something new.”*

*“It was exciting. I remember being happy that it happened on my shift. If it has to happen, then I would rather see it.”*

*“This time I was completely detached and because I did not have to tell the family. It was not my patient so just a guy who came to the ED.”*

Despite this, some students expressed guilt about feeling numb and detached and uncertainty about the proper reaction to have. It was also acknowledged that having no direct responsibility for the patient decreased the expected unpleasant feeling after the death. Increased exposure to death also had a similar effect.

Physical reactions to patient deaths were comparatively rare. These included an inability to sleep in some cases and a sensation of tingling in the hands.

*“It felt weird watching my hands because I had touched the person. I had a weird feeling in my hands. I felt they were tingling.”*

With regards to the cognitive aspect, thinking about the death and the idea of death seemed to be a recurring theme for some students. For more traumatic cases, the imagery of the patient death was remembered vividly and flashbacks were experienced when similar cases appeared.

*“For the first 3 hours I was just sitting there and thinking about what happened.”*

*“Sometimes I get flashbacks when I see a situation that reminds me of that moment.”*

Students commonly reported wanting to distance themselves and detach as a way to lessen the emotional impact; one student labeled this as a form of defense mechanism. A minority of students opted to talk to colleagues, friends and family, and ‘try to be involved in other things’ as coping methods. Another student stated that experiencing the worst helps in adapting to and coping better with future deaths.

*“I can't have emotion, too much emotion, or it will devastate me. So it made me try to protect myself and be more distant.”*

A large number of students (7) cited the ability to regulate emotions as a learning experience as well as a coping mechanism. Specifically, learning how to become less attached and expecting the worst. Students felt more prepared, confident and in some cases desensitized.

*“It severely grounded me form the beginning so now I’m prepared for the easier things.”*

Becoming more empathetic and gaining experience on how to deal with the family were also considered valuable learning experiences.

## Discussion

As expected, students expressed distress and sadness in both settings. The reaction and the extent of the effect was determined by contextual factors including the patient’s age, the expectation or suddenness of death, the first death experience, if the death was relatable to personal experiences, the extent of interaction with the patient and their families, the presence of a family reaction, and the coping mechanisms adopted. Students generally reported inpatient settings as more impactful. This was attributed largely to the high level of interaction and involvement with patients and their families that is normally not present in the ED. Cases in the ED, however, were categorized as impactful with some additional conditions: if the trauma and imagery was visibly disturbing and if the family reaction was emotionally moving.

Although there is some data on medical students’ responses to patient death in various inpatient settings, the present study illustrates that dealing with death in the ED for students produces reactions of a different nature, with the above-mentioned variables moderating this reaction. This is most visible in the spontaneous differences that emerge in the emotional labels expressed in each setting. Deaths in the inpatient setting elicited words such as grief, sadness, feeling heartbroken and upset. Deaths in the ED triggered reactions relating more to the suddenness that is common in the ED including: shock, confusion, surprise, and specific reactions to the imagery of trauma cases. As such, dealing with deaths in the ED may require or demand a different set of skills from the student. Debriefing and counseling sessions on ED deaths may also need to be tailored accordingly. In similar fields where traumatic deaths are often encountered, tailored interventions such as critical-incident stress debriefing (CISD), a widely used group intervention to reduce post-traumatic stress in high risk occupations, such as disaster response and fire fighting, has been introduced [19]. Future studies could assess similar debriefing interventions in the ED setting.

Another notable differentiating characteristic is the impact of the imagery of trauma cases seen in the ED. Students offered more vivid descriptions and recollections of these patients as a result. Remarks were most commonly made on the goriness and graphic nature of the image.

Students found patient deaths in the ED more impactful if they witnessed the family’s reaction to the death, even if no direct interaction took place. In one case, a student explains that watching the family’s reaction was more disturbing than dealing with the patient death itself.

Several explanations have been put forth to understand physician reactions to patient death [15]. One is that patient death forces physicians to confront their own mortality as well as reminders of their loved one’s. Cognitive theories of stress highlight that exposure to trauma may lead to questioning one’s basic assumptions and beliefs about the world. This has been shown in the current study, with more powerful emotional reactions reported when previous experiences are brought up. Other models explaining student reactions to death have presented the following phases: preparation, the event, the crisis, the resolution. As with this sample, preparation for the event was minimal. The event itself is determined by whether an old/young/unexpected death occurred, with the latter two conditions resulting in more difficult deaths for the physicians. The resolution is referred to here as coping mechanisms and could entail rationalizations, learning experience, and contemplation.

Notably, emotional reactions to death are influenced by culture. Individuals of non-western groups tend to view emotions as highly pertinent to beliefs, with emotions more readily causing belief change when compared to western cultures. Typically, non-western groups tend to endorse an interdependent self-construal, a self-concept which places higher value on relationships and emotional connections and is strongly shaped by the feelings of others [20, 21]. As such, the self is particularly attentive to emotions and motivations of others. Non-western groups in this context refer to parts of Asia, Africa and Central and South America. By contrast, individualist groups generally endorse independent self-construals, driven by internal attributes and separate from the influence of others.

Most students expressed an interest in either pre- or post-counseling to help manage death’s effects, with more preferring post-counseling or debriefing. Some argued that anticipating the emotions students might encounter is not possible and so pre-counseling will not be as effective. None of the students interviewed reported discussing a patient death or an emotional reaction with an attending.

### Emotional reactions and professional development

Appropriate emotional reactions to patient deaths and coping mechanisms are often implicitly shaped by the hidden or unofficial curriculum. This is transmitted through the immediate environment and culture of the institution, including its attitudes and values, ultimately influencing students’ professional development [22]. As evidenced by this study, death is a learning and potentially transformative experience for students. Reactions and experiences of death embedded in the hidden curriculum, however, can lead to burnout, exhaustion and “ethical erosion” in some cases, which negatively affects developing professionalism in physicians [23]. Students experiencing death in the ED may be an especially vulnerable group due to higher exposure to trauma and shock as presently described, rendering attention to the hidden curriculum especially important.

### Limitations

There were at least two interviewers in any given interview which may have increased the number of follow up questions asked, potentially confusing the interviewee. One of the interviewers was involved in teaching the students but all teaching and grading happened prior to the interview and after the end of their rotation thus attenuating significantly the possibility of a dependent relationship. Additionally, since the topic is sensitive, this may have prevented participants from sharing their emotions openly and honestly. Recall bias may have limited the generalization of the results further.

## Conclusion

The emotional impact of patient death on students in the ED was different to the inpatient setting as described. Future studies may wish to assess the long-term impact of ED deaths in comparison to deaths in an inpatient-setting. Importantly, developing and testing debriefing techniques and sessions to support students and physicians in the ED setting may be a beneficial next step.

## Additional file

Additional file 1: Semi structured interview guide. The semi structured interview guide includes the questions asked to participants. (DOCX 18 kb)

## Abbreviations

AUB: American University of Beirut
ED: Emergency Department
