# Supplementary material for: The effect of patient death on medical students in the emergency department
Source: BMC Med Educ. 2017 Jul 10;17:110. doi: 10.1186/s12909-017-0945-9 (PMC5504556; doi:10.1186/s12909-017-0945-9)
Supplement: Additional file 1: — Semi structured interview guide. The semi structured interview guide includes the questions asked to participants. (DOCX 18 kb) [file 12909_2017_945_MOESM1_ESM.docx]

**Semi-structured interview questions**

Good afternoon,

Thank you for taking the time to meet with me. I will honor your time by making sure that we wrap up in the next approximately 30 minutes. As indicated in the consent form, the interview will be tape recorded to allow transcription and analysis of the data at a later stage. No identifiers will be recorded or collected. The data collected will be kept confidential; no one will have access to the data other than the principal investigator and the research assistant working on the study. Your participation is voluntary; you can withdraw at any time during the interview. Any questions before we start?

**I-General information about the participant (Demographic characteristics)**

- Study ID: ______
- Age: ______Years
- Gender: 🞏 Male 🞏 Female
- Did you witness any patient’s death previously?

🞏No 🞏Yes, How many? __when______

- Did you witness any family members’ death?

🞏No 🞏Yes, when______

**II-Death of the patient in the ER/inpatient setting**

1. Please tell me about the deceased patient in the ER/inpatient setting

🞏 Patient’s age

🞏 The symptoms of which the patient was suffering

🞏 Any relationship with the deceased patient

🞏 The patient died before you have been involved in his/her care

🞏 Involved in the process of giving care to the patient

1. Please tell me about your immediate feelings (*the same day the death occurred*) with the death of the patient?
2. Please tell me about your current feeling (today) with the death of the patient?
3. Following the death, please describe any interaction between you and any of the family members of the patient
4. How did the rest of the medical team behave/react to the death of the patient?
5. How did the medical team’s behavior affect you?
6. In what ways has your relationship to other patients changed since you experienced the death of a patient?

🞏 Are you closer, more distant, or is the relationship unchanged? Please explain.

1. In what ways can an information-sharing and event processing session among peers (debriefing) be helpful in coping with your emotions after a patient’s death?
